# Spontaneous esophageal perforation within a hiatal hernia: A case report

**DOI:** 10.1016/j.ijscr.2022.107484

**Published:** 2022-08-13

**Authors:** Hidenori Maki, Seiya Inoue, Masakazu Goto, Takeshi Nishino, Takahiro Yoshida, Hiromitsu Takizawa

**Affiliations:** Department of Thoracic, Endocrine Surgery and Oncology, Tokushima University Graduate School of Biomedical Sciences, Tokushima, Japan

**Keywords:** Spontaneous esophageal perforation, Boerhaave's syndrome, Hiatal hernia, Transabdominal approach

## Abstract

**Introduction:**

Spontaneous esophageal perforation, also commonly referred to as Boerhaave's syndrome, is one of the most lethal diseases causing an acute abdomen. Though rare, emergent surgical intervention is often required and management can be various based upon the site of the perforation. This literature has been written in line with the SCARE criteria (Agha et al., 2020) [1].

**Presentation of case:**

A 76-year-old man presented with acute abdominal pain. Computed tomography (CT) revealed and an emergent esophagogastroduodenoscopy (EGD) was performed carefully, which revealed a 7 cm all-layer esophageal laceration in the left lower esophageal wall. In our case, a hiatal hernia was protruding into the mediastinum, and the perforation site was inside of it, but there was no invasion into the thoracic cavity, thus a transabdominal approach was performed without thoracotomy.

**Discussion:**

This type of esophageal perforation within a hiatal hernia is quite rare and provides a unique clinical challenge. In addition, A review reported the average length of spontaneous esophageal perforation to be around 2 cm while our case had a perforation with a length of 7 cm. We chose the combination of the simple suture with omental buttress and wide drainage, but a complete fundoplication was impossible due to its large size of perforation.

**Conclusion:**

We chose the open abdominal approach because the case had high inflammation, a hiatal hernia and possibility of retro-gastric perforation. However, MIS should have been considered first if a situation or human resources allow it.

## Highlights

• Esophageal perforation combined with a hiatal hernia is quite rare.
• Our case had about 7cm of perforation though it is around 2cm on average.
• We chose the trans abdominal approach due to the hiatal hernia.
• In some conditions, thoracotomy is not required, and it would be beneficial when considering the invasiveness.

## 1 Introduction

Spontaneous esophageal perforation, also known as Boerhaave's syndrome, has been recognized as an important thoracoabdominal emergent condition. Its mortality rate is estimated as high as at least 20 % [2]. However, its overall incidence is quite low (0.031 % / year) [3]. Surgical intervention is the mainstay of treatment for this condition, however, several reports have suggested successful managements with drainage or antibiotics alone [4]. There are a wide variety of management strategies depending on the acuity of the individual patient and the resources of the treating medical facility and staff. Generally, the left lower part of the esophagus is the most susceptible site to perforate due to its anatomical fragility, and once it perforates into the mediastinum or intrathoracic cavity, it can lead to critical complications, such as a mediastinitis, pericarditis or sepsis [5], [6]. Often times, thoracotomy is necessary for appropriate management. Interestingly, our case was concomitant with a esophageal hiatal hernia protruding into the mediastinum. Here we present our management of this unique clinical challenge using a transabdominal surgical approach. This literature has been written in line with the SCARE criteria [1].

## 2 Presentation of case

**Fig. 1:**
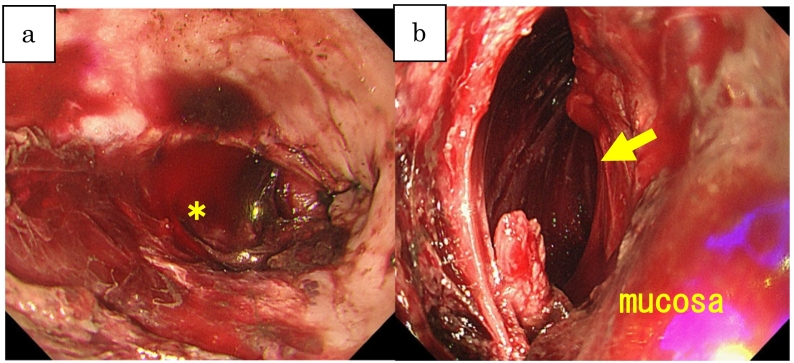
a: All layer rupture in the lower left wall (asterisk). b: Pseudo cavity external to the true esophageal lumen. Yellow arrow shows a pseudo cavity.

The patient was a 76-year-old man with a chief complaint of sudden repetitive hematemesis after lunch, followed by persistent epigastric tenderness. His past medical history was significant for bladder cancer and benign prostatic hyperplasia, for which he had completed the TUR-BT and chemotherapy. He presented to his local hospital where he underwent clinical evaluation followed by a non-contrast computed tomography (CT) of the chest, abdomen, and pelvis revealing a high-density area suggesting bleeding in his stomach. Upper gastrointestinal bleeding was suspected, so an emergent esophagogastroduodenoscopy (EGD) was performed. It revealed a 7 cm esophageal laceration extending into the muscle layer (Fig. 1-a), as well as a pseudo cavity between the mucosal and muscle layers in the left lower esophageal wall (Fig. 1-b). These findings were consistent with a spontaneous esophageal perforation. The patient was transferred to our department for further intervention.

**Fig. 2:**
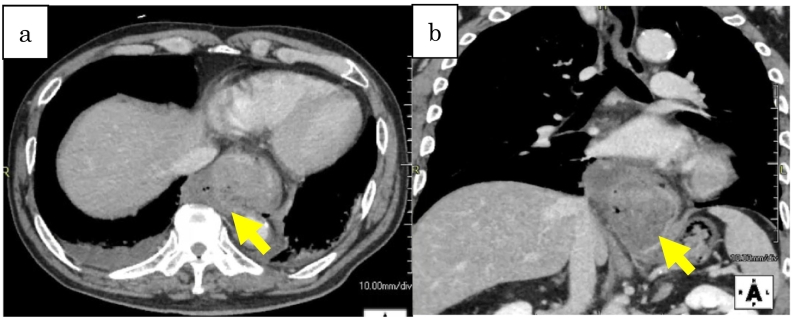
a: Dilated lower esophagus and free air around it. b: Huge hiatal hernia reaching as high as the pulmonary artery.

**Fig. 3:**
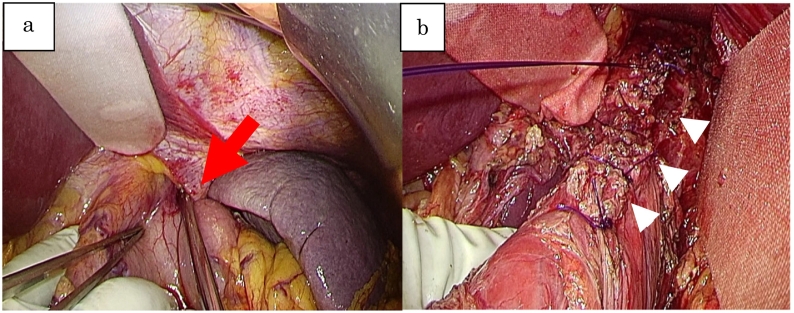
a: Incarcerated fundus of the stomach (red arrow). b: Suture site on muscle layer (white arrowhead).

He appeared in distress and had intense tenderness with guarding in the epigastric region when he arrived at our hospital. He was oriented for all, and vital signs are follows; Heart rate: 94 beats/min and regular, blood pressure: 164/111 mmHg, respiratory rate: 30/min, SpO2: 97 % (ambient air), and body temperature: 90.8 F. Laboratory data revealed as follows; WBC: 19,000/μL, C-reactive protein: 0.36 mg/dL, Hemoglobin: 12.8 g/dL, but otherwise were within normal limits. A new CT scan with contrast of the chest, abdomen, and pelvis was performed. This demonstrated lower esophageal dilation accompanied by fluid and free air around it. The abdominal CT scan demonstrated contrast extravasation from the stomach, further suggesting a perforation (Fig. 2-a). The hiatal hernia was detected in the coronal view, but there was no contamination of the thoracic cavity (Fig. 2-b). Combined with the findings by the previous EGD, spontaneous esophageal perforation was diagnosed. Emergent laparotomy was selected because there was no obvious invasion to thoracic cavity with the CT, in addition to the hiatal hernia seemed to be helpful to keep the view of the lesion. About 6 h had passed before the patient was rushed in to the theater since the diagnosis had been made, and 9 h passed after the onset. After the hernia contents were reduced into the peritoneal cavity. We detected a 7 cm, longitudinal, full thickness perforation to the lower esophagus. There was no damage to the gastric body. The site was contained within the hiatal hernia sac protruding into the mediastinum (Fig. 3-a). A two layered closure was performed to the ruptured mucosal and muscle layers respectively (4-0 PDS® and 3-0 PDS®) with the assistance of the intraoperative EGD (Fig. 3-b). An anterior cruroplasty was performed using permanent interrupted suture with 2-0 Proline®. A tongue of omentum was suture in place over the repair as a buttress. A leak test was then performed, which was negative, and the area was widely drained then a feeding jejunostomy tube was made. Enteral tube feeding was initiated two days after the surgery.

Unfortunately, 5 days after the index operation, inflammatory markers were trending up, and a leak was identified on an upper gastrointestinal fluoroscopy study with contrast. The decision was made to take the patient back to the operating room. During the second operation, we found an approximately 1 cm perforation at the most anal side of the closure and revision was impossible based on the friability of the tissue. Sufficient irrigation was applied followed by the replacement of the drainage tubes into hernia sac. On postoperative day (POD) 38, X-ray with oral contrast was performed which revealed no leakage then drainage tubes were removed. Water intake was resumed on the day. Oral food intake was restarted on POD 44 after confirming the complete healing of the site with repeat EGD. Finally, the patient moved to the original hospital for the physiotherapy 47 days after the first operation.

## 3 Discussion

Even normal esophageal perforation itself is comparatively rare, but a case accompanied with a hiatal hernia is extremely rare. We found only a few reports of similar cases while performing a literature review on Pub Med searching with the words “esophageal perforation” or “Boerhaave's syndrome”, and “hiatal hernia” within the time frame of 1952–2022 [7], [8]. Only one Japanese team reported a similar case treated with surgical repair in a domestic journal [9]. Esophageal perforation is roughly categorized into two types, which are exclusive in the mediastinum or perforating into the thoracic cavity. Considering there was no contamination into the thoracic cavity, we deem our case as a subtype of the former. A review reported the average length of spontaneous esophageal perforation to be around 2 cm. Cases of perforation of a length longer than 5 cm were found to be <10 %, while our case had a perforation with a length of 7 cm [10]. The combination of the huge tear and hiatal hernia make this case unique and particularly challenging. It is unclear whether or not the hiatal hernia contributed to the perforation, however, the hernia sac appeared to be protective, preventing contamination of the thoracic cavity and the resultant increased morbidity and mortality.

Based on the hypothesis that the hiatal hernia contributed to this perforation, it was likely due to a unique and/or unusual physiologic mechanism. Given the perforation site was located exclusively on the abdominal esophagus and not the lower thoracic esophagus in addition to the larger size of the tear, the greater intraabdominal pressure might be concentrated to the abdominal esophagus or the abdominal esophagus might be directly compressed by the fundus of the stomach within the hernia sac. To the best of our knowledge, only one similar case has been reported so far. It was also approached transabdominally and they also lacked clarity on the mechanism of perforation. While the etiology/pathophysiology in inconclusive, it is likely unique in respect to typical spontaneous esophageal perforations without hiatal hernia.

Fundic patch is also well-known method to strengthen the perforated site and prevent reflux post-operatively [11]. It was considered as one of the options during the primary surgery, but the tear was too large (7 cm) to perform it. Based on the Cameron criteria, abdominal esophageal perforation is the absolute indication for the surgical intervention, and some series have shown that esophagectomy could be considered if the perforation >5 cm in length due to the risk of leakage [12], [13].

Our patient clearly required surgical intervention, and esophagectomy might have been considered given the length of the lesion. However, it was challenging due to the location combined with the hiatal hernia, hence we chose the combination of the simple suture with omental buttress and wide drainage. In this case, leakage was observed from the repaired part of the lower esophagus after the primary surgery. One of the lessons learned from this experience is that it might be important to perform the fundic patch as much as possible even if covering the entire damaged site is challenging. Several reports mention that minimally invasive surgery (MIS) including laparoscopic, thoracoscopic or both of them could be performed safely for Boerhaave's syndrome, even if it is highly inflamed. They showed some MIS technique such as Toupet's partial fundoplication or omental buttress with flap, which reached preferrable consequences [14], [15]. On this case, we chose open abdominal approach given that possible retro-gastric perforation or huge perforation, however, when looked it back retrospectively, we should have considered the examination laparoscopic surgery first, then switched to open approach if MIS was impossible. Although one of the more serious complications from esophageal repair is post-operative leak, the greatest contributor to post-operative morbidity and mortality is delay between identification of perforation and intervention [12]. Fortunately, the patient was diagnosed correctly in the early phase of his care, which led to an overall favorable outcome.

This type of perforation is challenging, but possible to treat with a trans-abdominal approach as long as there is no contamination in the thoracic cavity. An abdominal approach may have the advantage of avoiding some of the morbidity associated with thoracotomy. We believe it was the right approach in this patient and perhaps the correct approach in patients with similar presentations in the future.

## Funding

There are no funding sources.

## Ethical approval

The ethical approval was given by our institution.

## Consent

Our patient and family consented.

## Author contribution

Hidenori Maki: Primary assistant of the surgery and corresponding author

Seiya Inoue: Primary surgeon of the surgery, supervisor and writer

Masakazu Goto: Assistant of the surgery and writer

Takeshi Nishino: Assistant of the surgery and writer

Takahiro Yoshida: Assistant of the surgery and writer

Hiromitsu Takizawa: Assistant of the surgery, supervisor and writer

## Registration of research studies

Not applicable.

## Guarantor

Hidenori Maki, M.D.

## Declaration of competing interest

We don't have no conflicts of interest to declare on this literature.
